# *XACT*, a long non-coding transcript coating the active X chromosome in human pluripotent cells

**DOI:** 10.1186/1756-8935-6-S1-O33

**Published:** 2013-03-18

**Authors:** Céline Vallot, Christophe Huret, Yann Lesecque, Alissa Resch, Noufϊssa Oudrhiri, Annelise Bennaceur, Laurent Duret, Claire Rougeulle

**Affiliations:** 1 Univ Paris Diderot, Sorbonne Paris Cité, Epigenetics and Cell Fate, Paris, France; 2 CNRS, UMR7216 Epigenetics and Cell Fate, Paris, France; 3 Laboratoire de Biométrie et Biologie Evolutive, UMR CNRS 5558, Université de Lyon, Université Lyon 1, Villeurbanne, France; 4 Stem Cell Institute, UCHC, Farmington, CT, USA; 5 INSERM U935, Université Paris Sud 11, AP-HP, Villejuif 94802, France

## 

X-chromosome inactivation (XCI), the dosage compensation process that equalizes X-linked gene expression between sexes, has mostly been studied in the mouse, where the central role for the non-coding RNA *Xist* in the initiation and spreading of the process was demonstrated. Although *Xist* is conserved in humans [1], very little is known concerning its regulation and function in this species. Several lines of evidence moreover suggest that different strategies have been adopted in the human to control XCI as compared to the mouse. In particular, in human pre-implantation development, *XIST* RNA coats the X chromosome(s) in both male and female embryos without inducing X-chromosome silencing [2]. This indicates that *XIST* expression and X-inactivation can be uncoupled during human embryogenesis and that other elements likely participate to the control of X chromosome activity in humans.

XCI is established early during embryonic development, and embryonic stem cells can be used to decipher the kinetics and the molecular actors of the process. Human female embryonic stem cells (hESC) can be found in different configurations regarding *XIST* expression: most female hESC have already undergone XCI but tend to spontaneously lose *XIST* expression [3]. In the course of an RNA-seq analysis of female hESC, we identified an extended and un-annotated transcribed region producing a long unspliced, likely non-coding nuclear RNA. RNA-FISH analysis reveals that this transcript is expressed from, and coats the active X chromosome. We called this transcript *XACT*, for X-active coating transcript. In female hESC in which *XIST* is repressed, *XACT* is expressed from and coats both Xs, and this correlates with significant reactivation of the inactive X chromosome. Expression of *XACT* appears to be specific for pluripotent cells as its expression decreases during differentiation. Finally, we provide evidence that *XACT* is not conserved in the mouse.

In conclusion, we have identified *XACT* as the first long ncRNA that coats the active X chromosome in human. Given its expression profile and lack of conservation, it is tempting to speculate that *XACT* is involved in the peculiar control of XCI initiation in human.
